# Modeling structure–activity relationships with machine learning to identify GSK3-targeted small molecules as potential COVID-19 therapeutics

**DOI:** 10.3389/fendo.2023.1084327

**Published:** 2023-03-06

**Authors:** Rameez Hassan Pirzada, Bilal Ahmad, Naila Qayyum, Sangdun Choi

**Affiliations:** ^1^ Department of Molecular Science and Technology, Ajou University, Suwon, Republic of Korea; ^2^ S&K Therapeutics, Ajou University Campus Plaza, Suwon, Republic of Korea

**Keywords:** GSK3, machine learning, coronaviruses, QSAR, molecular descriptors

## Abstract

Coronaviruses induce severe upper respiratory tract infections, which can spread to the lungs. The nucleocapsid protein (N protein) plays an important role in genome replication, transcription, and virion assembly in SARS-CoV-2, the virus causing COVID-19, and in other coronaviruses. Glycogen synthase kinase 3 (GSK3) activation phosphorylates the viral N protein. To combat COVID-19 and future coronavirus outbreaks, interference with the dependence of N protein on GSK3 may be a viable strategy. Toward this end, this study aimed to construct robust machine learning models to identify GSK3 inhibitors from Food and Drug Administration–approved and investigational drug libraries using the quantitative structure–activity relationship approach. A non-redundant dataset consisting of 495 and 3070 compounds for GSK3α and GSK3β, respectively, was acquired from the ChEMBL database. Twelve sets of molecular descriptors were used to define these inhibitors, and machine learning algorithms were selected using the LazyPredict package. Histogram-based gradient boosting and light gradient boosting machine algorithms were used to develop predictive models that were evaluated based on the root mean square error and R-squared value. Finally, the top two drugs (selinexor and ruboxistaurin) were selected for molecular dynamics simulation based on the highest predicted activity (negative log of the half-maximal inhibitory concentration, pIC_50_ value) to further investigate the structural stability of the protein-ligand complexes. This artificial intelligence-based virtual high-throughput screening approach is an effective strategy for accelerating drug discovery and finding novel pharmacological targets while reducing the cost and time.

## 1 Introduction

Coronaviruses are a family of enveloped viruses known for their ability to infect humans, typically leading to respiratory illnesses (1, 2). To address the current epidemic of the novel coronavirus SARS-CoV-2 causing COVID-19, numerous approaches for virus identification and infection prevention and treatment are required. In this context, high-throughput screening has been performed to identify bioactive compounds that inhibit SARS-CoV-2 replication in tissue culture models (3–6). However, the mode of action and clinical efficacy of these candidates remain to be fully characterized, and additional targets need to be identified to further combat new and emerging SARS-CoV-2 variants. Among other structural and non-structural SARS-CoV-2 proteins, the nucleocapsid (N) protein is an essential RNA-binding protein that plays a crucial role in viral replication, transcription, and assembly (7–11). Inhibiting SARS-CoV-2 transcription will be a crucial objective, along with strengthening the immune response to the virus and reducing cytokine release syndrome linked to severe cases of COVID-19.

Glycogen synthase kinase 3 (GSK3) is a serine-threonine kinase signaling protein that plays a crucial role in a variety of biological processes, and its aberrant activity has been associated with diabetes, inflammation, and neurodegenerative and psychiatric disorders (12–15). GSK3 has two structurally identical isoforms (α and β), which are 97% similar in their catalytic domains but differs in their N and C (16–18). Both of these isoforms shows 98% of sequence identity (19). GSK3 (α and β) are required for the phosphorylation of SARS-CoV-2 N protein, and its inhibition blocks SARS-CoV-2–mediated infection in human lung epithelial tissues (16). In addition, knockout of GSK3α and GSK3β validates that it is vital for N phosphorylation (16). Inhibition of GSK3 can increase adaptive T cell and innate natural killer responses of CD8^+^ T cells while also inhibiting SARS-CoV-2 viral replication (20). Moreover, GSK3 phosphorylates N proteins within the arginine-serine (RS) region of the JHM strain of mouse hepatitis virus (JHMV) and SARS-CoV, which caused the outbreak of severe acute respiratory syndrome in 2002–2004 (9, 10, 21, 22). Phosphorylation of the JHMV N protein is necessary for the recruitment of the ATP-dependent RNA helicase DDX1 for the transcription of long sub-genetic RNAs (10). The N protein from infectious bronchitis virus (IBV) and SARS-CoV interact directly with GSK3, and its knockdown was shown to disrupt the replication of IBV in the Vero cell line (23, 24). Moreover, GSK3 inactivators inhibit the coronavirus protease Mpro (or 3C-like protease), which cleaves SARS-CoV-2–encoded polyproteins (pp1a and pp1ab) required for viral replication and transcription. Additionally, GSK3β was also identified to control the autophagy pathway as it involves in the regulation of transcription factor EB (TFEB) nuclear expression mediated *via* mechanistic target of rapamycin complex 1 (mTORC1) dependent manner (25, 26). It also modulates TFEB through the signaling pathways of protein kinase C (PKC) and eukaryotic translation initiation factor 4A-3 (eIF4A3) (27, 28). Furthermore, GSK3β-induced phosphorylation of TFEB leads to its cytoplasmic retention and contributes to the blockage of the lysosomal-mediated autophagy pathway (29, 30). Autophagy plays a critical role in the degradation of dysfunctional cytoplasmic organelles and infectious pathogens, whereas defective autophagy has been observed in SARS-CoV-2 pathogenesis (31, 32). An interplay between viral infection and autophagy directs towards the development of an effective therapeutic approach for COVID-19 (31, 33). Therefore, it is reasonable to propose that inhibition of GSK3 using small-molecule inhibitors would effectively block SARS-CoV-2 replication as found for SARS-CoV-1.

Quantitative structure–activity relationship (QSAR) approaches are increasingly being applied to aid in the preclinical development of small-molecule drugs. QSAR models help predict the physicochemical properties, biological activity, toxicity, chemical reactivity, and metabolism of chemical compounds (34–36). The main goal of QSAR analysis is to link a set of predictor variables (X) to the response variable (Y). Techniques for linking X and Y and molecular descriptors have received substantial research attention. In this context, a key strategy in drug discovery is the development of machine learning (ML) techniques to estimate drug-target interactions. QSAR approaches employ a variety of linear and non-linear ML algorithms to produce predictive models for ligand binding to a biological receptor. The term “QSAR” refers to regression models that establish quantitative relationships between molecules’ chemical structures and their physical, chemical, or biological characteristics. ML techniques such as gradient boosting, support vector machines, partial least squares, artificial neural networks, or linear regression use a set of molecular descriptors as input data to predict chemical features.

We attempted to develop ML-based QSAR models that could identify GSK3 inhibitors using the bioactivity data available in the ChEMBL and PubChem databases. ML models were developed using two algorithms, histogram-based gradient boosting (HGBM), and light gradient boost machine (LGBM), to prospectively identify GSK3 inhibitors from the Food and Drug Administration (FDA)–approved drug library. The rationale behind selecting GSK3 as a drug target is that most of the anti-viral therapies are primarily designed to target the viral structure which however is frequently associated with drawbacks such as drug resistance as a consequence of viral mutation (37, 38). Here drug-oriented machine learning-based repurposing approach was adopted based on the physicochemical and pharmacological properties of both active and inactive GSK3 inhibitors to build a model that can identify already approved drugs against the selected target (GSK3). Furthermore, this drug discovery strategy is highly efficient, saves time, and cost and proves to be a prospective approach towards finding already approved drugs against SARS-CoV-2 (39). Finally, molecular dynamics (MD) simulation was performed to further investigate the structural stability of the protein-ligand complexes.

## 2 Materials and methods

### 2.1 Data compilation and curation

**Figure 1:**
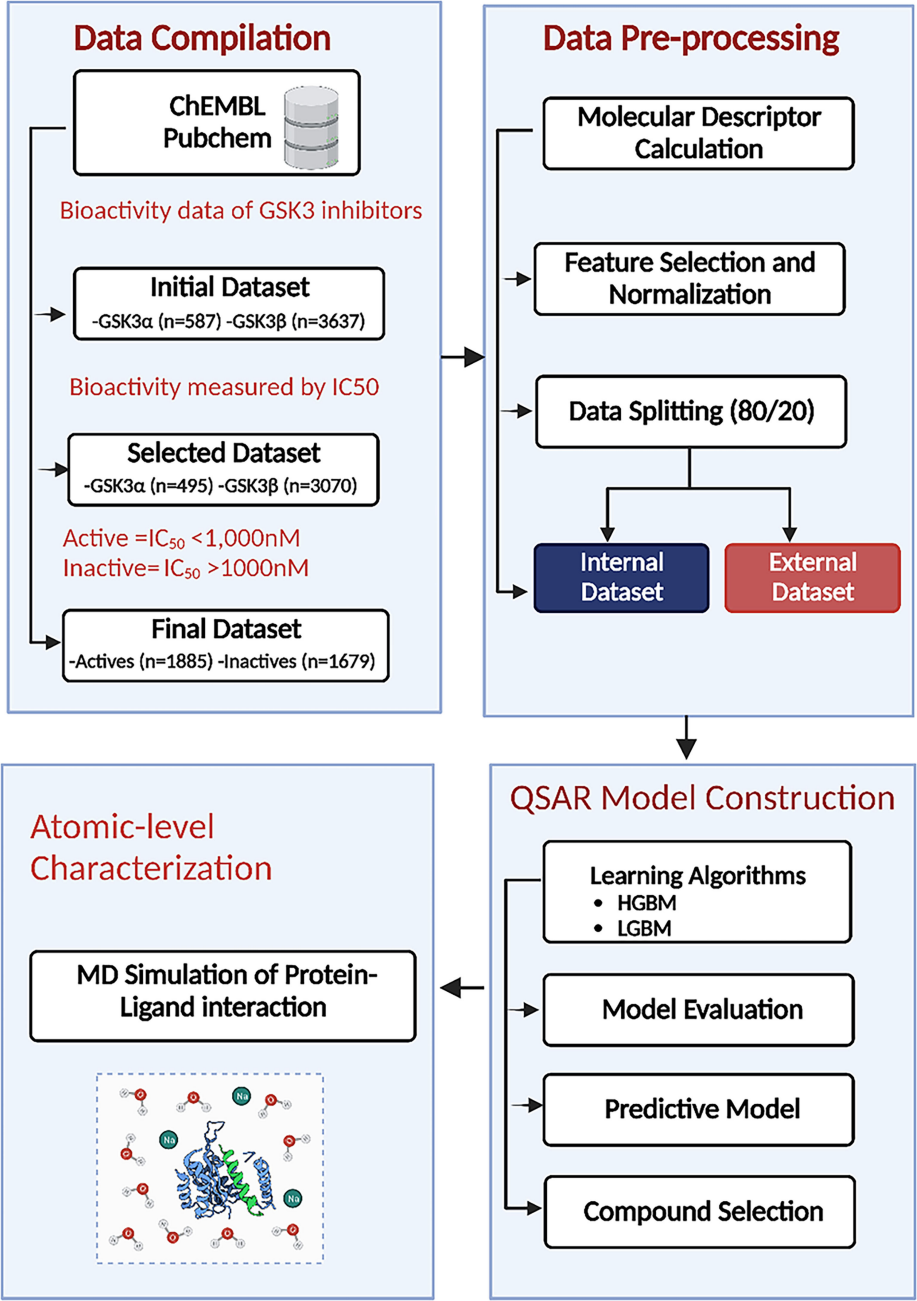
Machine learning (ML)–based quantitative structure–activity relationship (QSAR) and structural dynamics analysis workflow. HGBM, histogram-based gradient boosting ML model; LGBM, light gradient boosting ML model.

The GSK3α (Target ID: CHEMBL2850) and GSK3β (Target ID: CHEMBL262) datasets used in this study were extracted from the ChEMBL database to generate ML models (40). These datasets are composed of a diverse set of molecules that have been experimentally validated for their inhibitory activity against GSK3. A schematic illustration of the ML-based QSAR and structural dynamics workflow used in this study is shown in **Figure 1**. Initially, the total number of collected compounds with various bioactivity units, including IC_50_, Ki, EC_50_, and KD, were collected for GSK3α and GSK3β, consisting of 587 and 3637 molecules, respectively. The dataset was cleaned and preprocessed by applying the following filters: first, compounds with undefined activity were discarded; second, compounds containing salt or mixtures, along with overlapping compounds, were removed. Furthermore, in this study, the subset of bioactivity data obtained with IC_50_ as a unit was investigated for both GSK3α and GSK3β, consisting of 495 and 3070 unique bioactive compounds, respectively. As the objective of this study was to develop classification models of biologically active compounds, the activity dataset was divided into active and inactive compounds with IC_50_ thresholds of<1 and >10 μM, respectively, whereas compounds with intermediate activity levels were not considered. The selected compounds were filtered based on Lipinski’s rule of five (RO5). Finally, after pre-and post-processing, two sets of non-redundant and curated compounds for GSK3 (α, β) were used for further investigation: 1885 active compounds and 1679 inactive compounds. All of the collected compounds were randomly segregated into a training set to generate the QSAR classification model and a test set to validate the model quality with a ratio of 80:20.

### 2.2 Molecular descriptors

A molecular descriptor is a mathematical (quantitative/or qualitative) representation of a molecule that is encoded with various sources of chemical information, which is converted and coded to deal with the biological, chemical, and pharmacological features of different small molecules that are used for model construction (41, 42). To develop robust non-linear binary QSAR classification models with better performance, various descriptors such as electronic, topological, geometrical, and spatial descriptors were computed for each molecule in the dataset. We used PaDEL-Descriptor software for molecular descriptor calculation based on a Python code to calculate 12 sets of molecular descriptors, as shown in **Table 1** (43). The descriptors belong to nine different types: CDK fingerprint, CDK extended, CDK graph only, Klekota-Roth, AtomPairs 2D, MACCS, E-state, PubChem, and Substructure. These descriptor types can be further divided into two versions: binary and count versions. In this context, the descriptors Klekota-Roth, AtomPairs 2D, and Substructure belong to both versions and provide a detailed description of the substructural components of the studied molecules. The remaining descriptors (n = 9) belong to the binary version. We also computed Lipinski’s RO5 molecular descriptors to be used as classification parameters for the identification of drug-like molecules.

**Table 1:** Calculated using the PaDEL-descriptor, 12 sets of fingerprint descriptors.

Fingerprint	Abbreviation	Number (bits)	Fingerprint Pattern Type	Description
CDK	FP	1024	Hash fingerprints	Fingerprint of length 1024
CDK extended	ExtendedFP	1024	Hash fingerprints	Adds fingerprint information about ring features.
CDK graph only	GraphOnlyFP	1024	Hash fingerprints	A distinct approach that considers connectivity and ignores bond order
MACCS	MaccsFP	166	Structural features	Chemical characteristics represented in binary using MACCS keys
Substructure	Substructure	307	Structural features	SMARTS patterns for functional groups are present.
Substructure count	nSubstructure	307	Structural features count	SMARTS patterns counted for functional groups
2D atom pairs Count	nAP2DC	780	Structural features count	Atom pair count at different topological distances
2D atom pairs	AP2D	780	Structural features	Atom pair presence at different topological distances
PubChem	PubChemFP	881	Structural features	Binary representation of the PubChem-defined substructures
Klekota–Roth	KRFP	4860	Structural features	Existence of chemical substructures
Klekota–Roth Count	nKRFP	4860	Structural features count	Substructure count for chemicals
E-state	EstateFP	79	Structural features	Types of atoms with respect to electrotopology

### 2.3 Data filtering

Molecular fingerprints with redundant or constant variables were discarded to remove any inherent biases that could negatively impact the resulting model. Not all molecular descriptors are required to represent the features of inhibitors and non-inhibitors. Moreover, the model learns the biases in the data and continues to amplify them, which could lead to overfitting. A selection criterion is required to discard irrelevant descriptors that can measure the relevance of a specific descriptor to the output of any classifier (44). In this context, the VarianceThreshold class from Scikit-learn was implemented to remove the low-variance features with a threshold value higher than 0.1, and the remaining features were used for further analysis.

### 2.4 Data splitting and test selection

**Figure 2:**
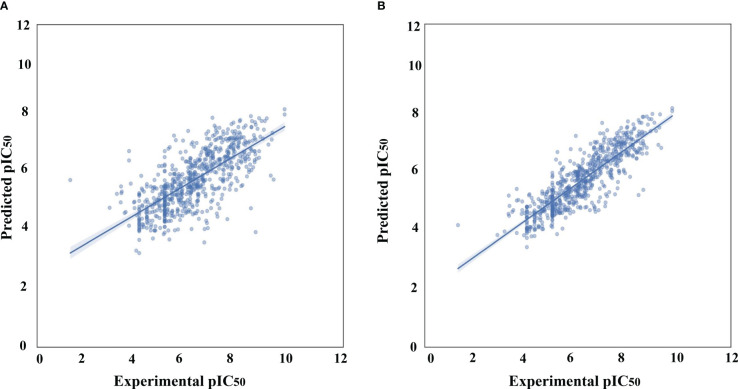
Correlation plots of experimental vs. predicted pIC_50_ values for GSK3 inhibition to the training and test set. **(A)** Histogram-based gradient boosting (HGBM) model. **(B)** Light gradient boosted machine (LGBM) model.

Following data filtering, the GSK3 (α, β) datasets were split using the Kennard–Stone algorithm, which separated the entire dataset into internal and external sets with a ratio of 80% and 20%, respectively. The internal datasets were used for training the ML model, and its capability to extrapolate to unknown molecules was simulated by analysis against the external dataset. Finally, the training set was used to estimate the performance of the model using a five-fold cross-validation scheme. The correlation plots for the experimental versus predicted pIC_50_ values for GSK3 inhibition in the training and test sets are shown in **Figure 2**.

Additionally, the effectiveness of the ML model was evaluated using 20 compounds that were identified as inhibitors of this target in numerous previous studies (45–47). The model performance measures and the activity threshold for this external dataset were compared with the experimental IC_50_ values, as shown in the **Supplementary Tables S1**, **S2**.

### 2.5 ML-based QSAR classification

The QSAR classification model can represent molecular descriptors as a relationship between the dependent variable (IC_50_) and independent variables (molecular descriptors), each demonstrating the category of the corresponding sample (GSK3 inhibitory activity). In structure–activity relationships, the association between the corresponding datasets is complex and non-linear; thus, a QSAR modeling–based approach was used because it has previously shown outstanding performance in this regard (48). In this context, ML algorithms can cluster instances or observations present in data into classes. A variety of ML algorithms have been employed to construct QSAR classification models from dataset activity labels and molecular descriptors. For example, support vector machines, naïve Bayes classifiers, neural networks, rule-based classifiers, and decision trees are various ML-based techniques used to elucidate the classification problem. In this study, the LazyPredict package was employed using a Python script for model selection, which generates a variety of ML algorithms and authenticates the best-performing algorithm, as shown in **Supplementary Table S3** (49). The top models were selected based on the R-squared and root mean squared error (RMSE) values to train our regression model to precisely predict the activity of GSK3 inhibitors. These ML algorithms were implemented using Python software. Subsequently, to determine the optimal values, hyperparameter tuning of the selected models was performed using GridSearchCV implemented in Scikit-learn. The list of the best parameters selected for hyperparameter tuning is presented in **Table 2**.

**Table 2:** The best parameters of machine learning algorithms following parameter adjustment using GridSearchCV.

Machine learning methods	Tuning parameter	Model performance R^2^
HGBM	n_estimators: 800 random_state: 100 learning_rate: 0.1 subsample: 1.0	0.72
LGBM	n_estimators: 100 max_depth: 9 learning_rate: 0.1 gamma: 0.1	0.70

### 2.6 Statistical assessment for model validation/performance

Model validation is a crucial step to ensure that a fitted model can accurately predict responses to unknown data. We used two statistical parameters, the Pearson correlation coefficient (r) and RMSE, to assess the performance of the QSAR models. The Pearson correlation coefficient is a common statistic used to describe the strength of the relationship between two variables of interest, which ranges from –1 to +1, with negative values denoting a negative correlation between two variables and positive values indicating a positive correlation. The relative error of the prediction model is frequently examined using the RMSE.

The Y-scrambling test, external validation, and 10-fold cross-validation were used to confirm the predictive capacity of the QSAR model. The 10-fold cross-validation method does not use the entire dataset to create a predictive model. As an alternative, it separates the data into training and testing datasets, enabling the model to be tested on the testing dataset using the training dataset as a basis. By repeating the 10-fold validation, the average accuracy was used to examine the performance of the prediction model. To further assess the performance and prediction accuracy of the model on external/benchmarking datasets, mean absolute percentage error (MAPE) was evaluated, a smaller value indicates better model performance.


$$\;MAPE=\frac{1}{N}\sum_{K=1}^{N}\frac{|X_{k}-{\hat{\text{X}}}_{k\;}|}{X_{k}}\;\times \;100\%$$

Xk is the actual value, X̂k is the predicted value of the model, |*X*_*k*_−X̂_*k* _| represents the absolute error, and N is the number of incidence data points.

### 2.7 Docking and MD simulation

The selected FDA-approved and natural product compounds were selected based on the predicted IC_50_ values produced by our QSAR model and docked to the active-site of GSK3 using a protocol described in our previous report (50). From the top 10 docked conformations the best pose was selected based on the MOE docking score (S), protein-ligand interaction fingerprint (PLIF) calculation, including hydrogen and ionic interactions. These selected best poses of selinexor, ruboxistaurin alongwith GSK3 apo and PF-04802367 (PDB ID: 5K5N) were subjected to MD simulation (200 ns) using GROMACS. Furthermore, the dynamic behavior and stability of each system was validated. The detailed methodology adopted here can be found in our previous study (50).

### 2.8 Binding free energy calculations

Molecular mechanics Poisson–Boltzmann surface area (MMPBSA) method was used to measure the binding free energy between GSK3 and ligand complexes. The Poisson-Boltzmann equation was used to determine the effects of the solvent’s polar and nonpolar components on the free energy.


$$\Delta G_{bind=}G_{complex}-\left(G_{receptor}+G_{ligand}\right)$$

$$\Delta G_{bind=}\Delta E_{MM\;}+\;\Delta \Delta G_{sol}-T\Delta S$$

In the above equation, ΔGbind represents binding free energy, ΔEMM is the intermolecular energy difference, ΔΔGsol shows the difference in solvation energy, whereas T and ΔS stands for absolute temperature and change in entropy. The study was performed using the gmx_MMPBSA (51). Frames were extracted throughout the trajectory with an interval of 30-frames. The detailed protocol is described in our previous study (52).

## 3 Results and discussion

The computational workflow for elucidating the underlying basis of the bioactivity of GSK3 is summarized in **Figure 1**. To gain a deeper understanding of the dataset, a standard chemical space analysis was performed on the investigated compounds. The preprocessed dataset was utilized to create predictive classification and regression models using the HGBM and LGBM models after rigorous data curation. Subsequently, hyperparameter optimization was performed to determine the optimal parameter configuration of the model. The best-performing model was used to evaluate the predictive capability after training the selected algorithms to gain biological insight. Finally, MD simulations were performed on selected FDA-approved drugs based on the pIC_50_ value to further assess the structural dynamics and stability of the protein–drug complexes.

### 3.1 Exploratory chemical space analysis of GSK3 inhibitors

A set of 3,565 compounds tested against GSK3 (α, β) were extracted from public databases. This dataset included seven bioassay formats characterized using the BioAssays Ontology number (53). However, 95% of the dataset was linked to the same bioassay (BAO_0000357) connected to the single-protein affinity format, which could infer the homogeneity of the dataset.

Chemical space analysis of GSK3 inhibitors was explored using Lipinski’s RO5 descriptors to gain an understanding of structure–activity relationships. Chemical space analysis can provide considerable understanding of the general characteristics of compounds that define their inhibitory activity. Exploratory data analysis was performed using RO5 descriptors, including the number of hydrogen bond acceptors (nHBAcc), number of hydrogen bond donors (nHBDon), molecular weight (MW), and octanol/water partition coefficient (LogP). The MW of a chemical compound is often used to compute its size, as it facilitates the analysis and prediction of the appropriate size of a drug, which is critical for its transport across a lipid membrane (54). Molecular hydrophobicity (lipophilicity) is usually computed as LogP, which is an important estimator of chemical membrane penetration and permeability (55). Moreover, nHBDon and nHBAcc were computed to estimate the hydrogen bond-forming capacity of a chemical compound.

Initially, the analysis was carried out by visualizing the distribution of active and inactive compounds as determined by the scatter plot of MW vs. LogP, followed by a comparative analysis of active and inactive compounds as a function of Lipinski’s RO5 descriptors (56).

**Figure 3:**
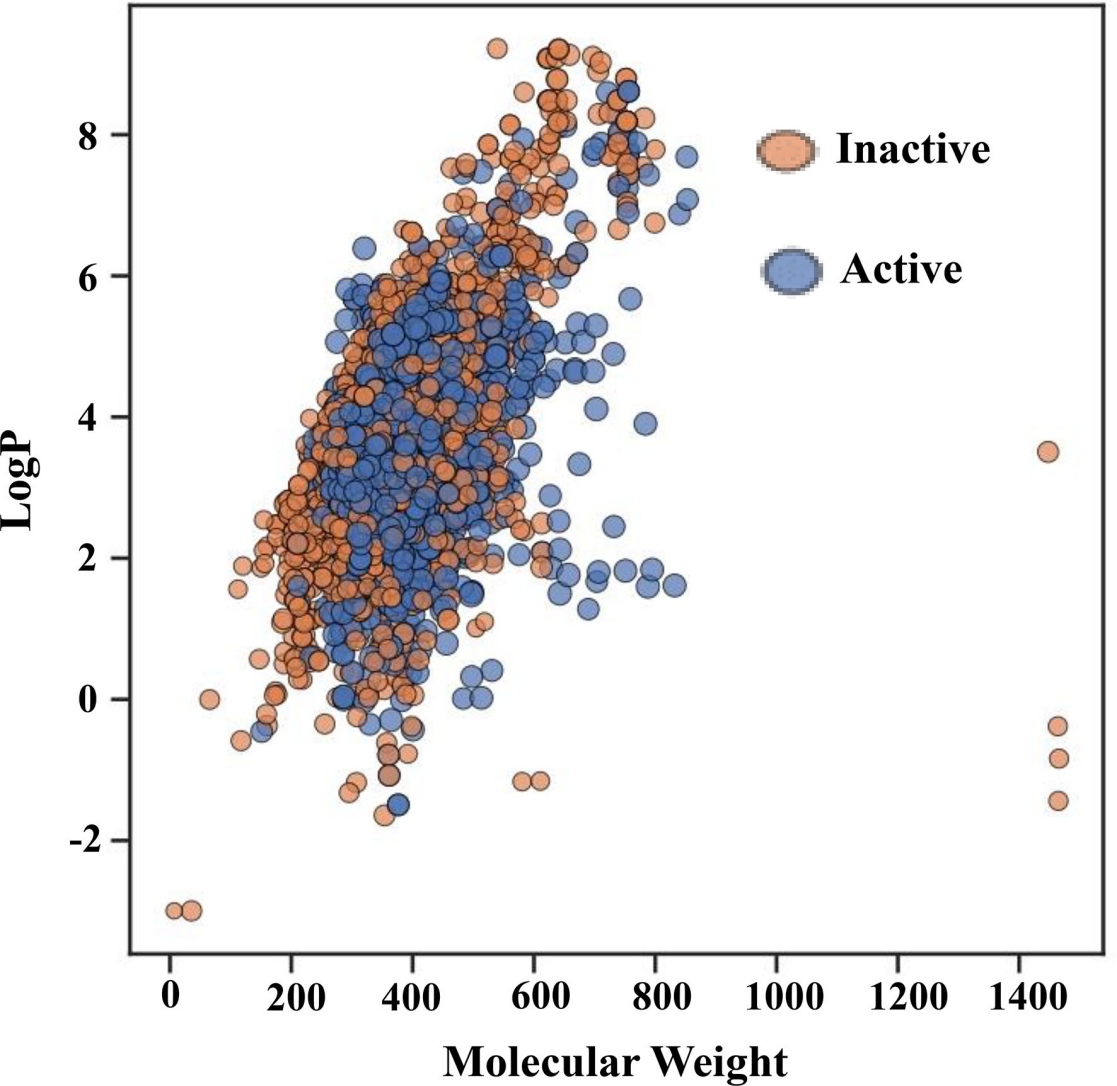
Chemical space of the training set. The molecular weight (MW) on the X-axis and the logarithm of the octanol/water partition coefficient (LogP) on the Y-axis serve as the parameters for the chemical space. Red and green spots, respectively, represent active and inactive substances.

**Figure 3** shows the chemical space distribution of the training set in a scatter plot of the MW versus the logarithm of the LogP. The MW and LogP of the active and inactive compounds showed almost identical distributions, with the majority of the compounds having an MW falling between approximately 200 and 600 Da and a LogP falling between 0 and 7. Most oral drugs are more likely to have optimal physicochemical and absorption, distribution, metabolism, and excretion properties between 1 and 4 (57, 58), which is also evident in **Figure 3**. Furthermore, **Figure 3** shows that MW and ALogP cannot be used to discriminate between active and inactive compounds because of their shared chemical space.

**Figure 4:**
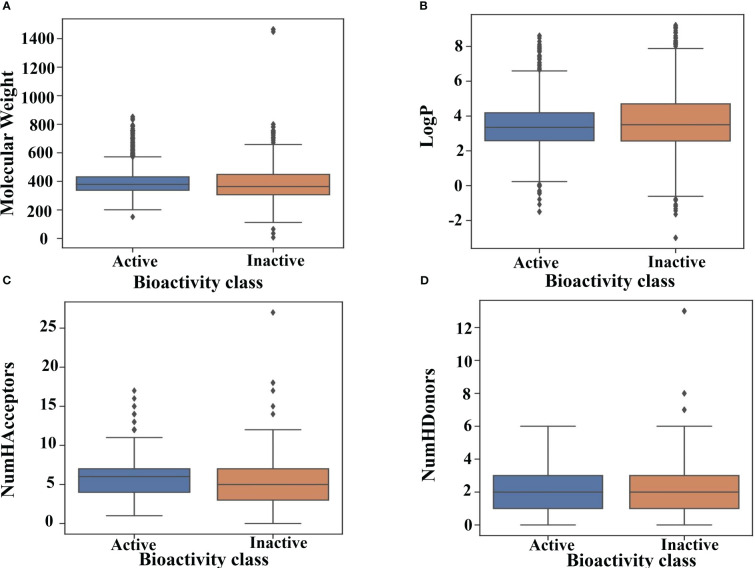
Drug-likeness evaluation. Box plot of GSK3 inhibitors using Lipinski’s rule of five descriptors. **(A)** molecular weight **(B)** logP **(C)** hydrogen bond acceptors and **(D)** hydrogen bond donors.

**Figure 4A** illustrates the total distribution of the compounds. As can be inferred from the median values in the box plot, active compounds tended to have a slightly higher MW of approximately 380 Da than that inactive compounds, which was approximately 350 Da. Similarly, inactive compounds had LogP values of approximately 3.8, which was higher than that of active compounds approximately 3.5 (**Figure 4B**). The distribution of nHBAcc, as deduced from the median, shows that the active compounds have a higher number of hydrogen bond acceptors (n ≈ 7) than inactive compounds (n ≈ 5) (**Figure 4C**). However, no significant differences were observed between the active and inactive compounds for nHBDon (**Figure 4D**). Therefore, it is difficult to predict the activity of inhibitors using simple molecular descriptors.

### 3.2 Model construction for the prediction of GSK3 kinase inhibitors using ensemble boosting

**Figure 5:**
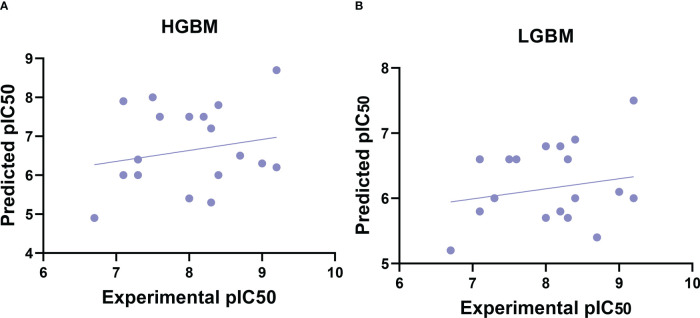
Correlation plots of experimental vs. predicted pIC_50_ values for GSK3 inhibition to the benchmarking dataset. **(A)** Histogram-based gradient boosting (HGBM) model. **(B)** Light gradient boosted machine (LGBM) model.

**Figure 6:**
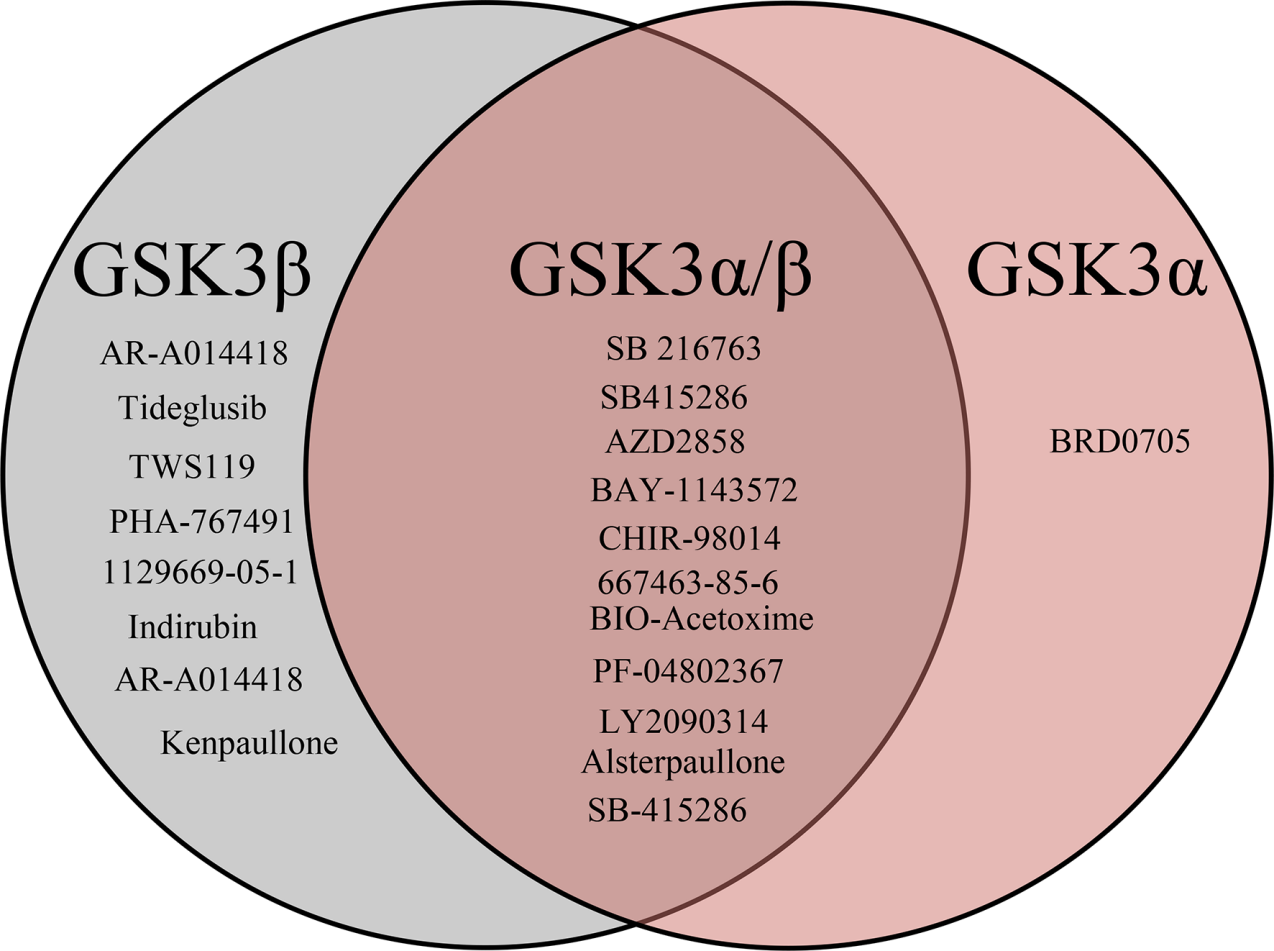
Venn diagrams visualizing the overlap of known GSK3 (α, β) inhibitors retrieved from the ChEMBL database. In total benchmarking dataset has 20 compounds out of which 8 are specific to GSK3β, 11 are to both GSK3 (α,/β) and 1 is to GSKα specific.

**Figure 7:**
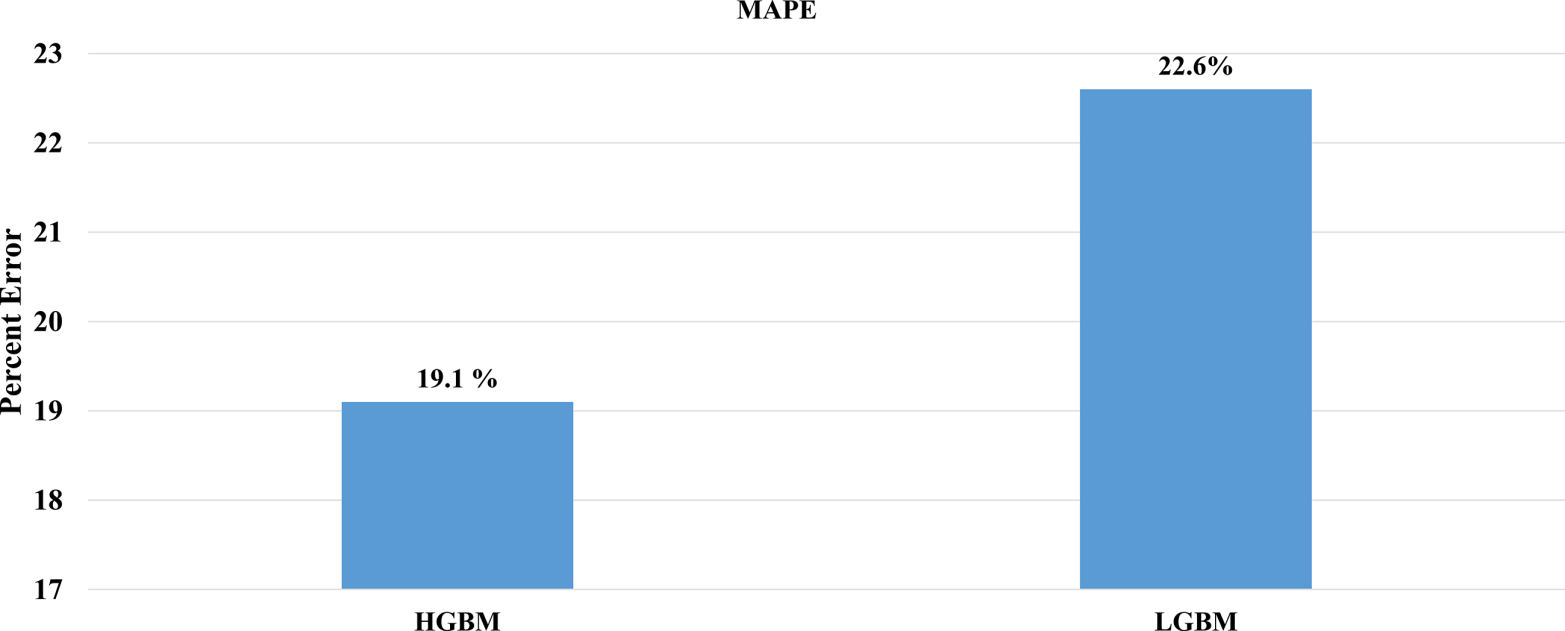
Comparison of mean absolute percentage error of the external test set. **(A)** HGBM and **(B)** LGBM in the context of prediction accuracy.

After the molecular descriptor calculation (**Table 1**), the LazyPredict package was used to acquire robust ML models, as described in Section 2.5. The best-performing models, HGBM and LGBM, with an R-squared value of 0.53 and 0.52 were used for model construction to better target the GSK3 kinase protein.The performance of the model was evaluated using the R-squared and RMSE metrics, as shown in **Supplementary Table S3**. Boosting algorithms (HGBM and LGBM) are a type of ensemble learning technique that gradually add tree models to fix the prediction error that already exists in the ensemble (59). To evaluate the performance of our ML models, an external test set using 20 known GSK3 inhibitors was used (45–47, 60). Because these external test set compounds were not considered when creating the models, the resulting performance showed the ability of the ML models to precisely predict the inhibitory activity of already known GSK-3 kinase inhibitors, as shown in **Supplementary Tables S1** and **S2**. The performance of the predictive ML model can also be seen in the correlation plot between predicted values vs. experimental values, as shown in **Figure 5**. Additionally, the overlapping GSK3 α, and β compounds in the benchmarking dataset of already known inhibitors are shown on **Figure 6**. The model evaluation metrics, MAPE was used to validate the prediction accuracy in benchmarking datasets (**Figure 7**). Both the HGBM and LGBM showed relatively good mean absolute percentage error scores of 19.1% and 22.6%, respectively, which represents the percentage difference between the predicted and experimental values. In addition to it, we compared the predictive performance of our QSAR model with another dataset (**Supplementary Table S4**). The data shows that compounds with higher activity in our model was comparable to the reported model (60).The results in **Table 2** indicate that both models exhibited good overall prediction accuracy; however, HGBM was the more accurate model.

This QSAR-based model was then used to predict the inhibitory activity of FDA-approved drugs against GSK3 kinase. Each compound was assigned a pIC_50_ value; higher values indicate that the drug is effective at lower concentrations against GSK3 kinase and therefore will show lower systemic toxicity (61).

The top 10 FDA-approved drugs based on pIC_50_ values from both the HGBM and LGBM models are presented in **Tables 3**, **4**. The drugs with the best-predicted pIC_50_ values produced by our model were selinexor and ruboxistaurin hydrochloride.

**Table 3:** GSK3 inhibition activity prediction by HGBM.

	Investigational and FDA Drugs	PubChem ID	pIC_50_
**1**	Selinexor	71481097	9.4125746
**2**	Raltegravir potassium	23668479	9.175947616
**3**	Dasabuvir	56640146	9.001651838
**4**	Kuvan (sapropterin)	135409471	8.848548463
**5**	Deferiprone	2972	8.171706964
**6**	Propylthiouracil	657298	8.399683851
**7**	Trelagliptin	15983988	8.202770399
**8**	Urapidil	5639	7.831437785
**9**	Ruboxistaurin	9870785	7.419813853
**10**	Methylcobalamin	10898559	6.098851787

pIC_50_ is the negative log of the IC_50_.

Histogram-based gradient boosting machine learning (HGBM).

**Table 4:** GSK3 inhibition activity prediction by LGBM.

	Investigational and FDA Drugs	PubChem ID	pIC_50_
**1**	Ruboxistaurin	9870785	7.448867965
**2**	Methylcobalamin	10898559	7.229432941
**3**	Cefpirome	5479539	7.050002608
**4**	Allopurinol	135401907	7.030920707
**5**	Simeprevir	24873435	7.007010834
**6**	Neratinib	9915743	7.000923602
**7**	Selinexor	71481097	6.96201482
**8**	Lafutidine	5282136	6.882663164
**9**	Enoxacin	3229	6.831823482
**10**	Cefodizime	5361871	6.828905555

pIC_50_ is the negative log of the IC_50_.

Light gradient boosting machine learning algorithm (LGBM).

Selinexor is an FDA-approved drug for the treatment of multiple myeloma that binds to and inhibits exportin-1 (XPO) and is being evaluated against SARS-CoV-2 in a phase-2 clinical trial (NCT04349098) (62). XPO-1 protein plays an important role in the export of RNA transcripts and nuclear proteins having leucine-rich nuclear export signals (NES) (47, 60–62). However, blocking XPO-1 with its selective inhibitors causes the nuclear accumulation of transcription factor EB (TFEB) and results in autophagy enhancement in human cells and model organisms (33). Because of this, it has been demonstrated that selinexor inhibits the spread of SARS-CoV-2 by preventing the movement of nuclear proteins into the cytoplasm (63, 64). Similar phenomena have been observed when GSK3 is inhibited; this causes translocation of TFEB into the nucleus, where it controls the transcription of around 400 genes involved in autophagy, which eliminates the invading viruses like SARS-CoV-2 (65–67). The current study indicates additional, mechanisms through which selinexor could prevent SARS-CoV-2 replication, such as by preventing the phosphorylation of the virally encoded N protein. In addition to the drug’s effect on autophagy (16, 20).

Ruboxistaurin is an investigational drug that targets protein kinase C beta for the treatment of diabetic retinopathy. Protein kinase C (PKC) is a serine/threonine protein kinase that has been identified to modulate autophagy (68). Autophagy is an innate immune response to kill and degrade invading viruses (69). In this context, one such mechanism is the activation of PKC-α/δ induced by GSK3β inhibition which leads to the phosphorylation repression of TFEB and its nuclear localization and activation of autophagy pathways (30, 70). The nuclear localization of TFEB induced by PKC-α/δ occurs *via* GSK3β in mTORC1-independent manner (30). Additionally, an orally active PKC inhibitor ruboxistaurin proves to be active against SARS-CoV-2 as it inhibits NETosis, and has passed phase 3 for other indications (71). Previous studies have also shown that ruboxistaurin was active against GSK3α and GSK3β with IC_50_ values of 695.9 nM and 97.3 nM, respectively (60). This data indicates that ruboxistaurin inhibits both PKC and GSK3 which supports our QSAR model prediction.

In conclusion, the top predicted medications (selinexor, ruboxistaurin) by our model also correlate with the data that revealed their effectiveness against SARS-CoV-2 as shown above, and GSK3 inhibition seems to play a significant part in the activity of these drugs. Furthermore, the drugs with the highest predicted IC50 including selinexor and ruboxistaurin indicate that the molecular fingerprints of these compounds exist in the compounds used to develop the training set of GSK3 inhibitors. In addition, we validate the performance of our ML model from a structural viewpoint, these two drugs were subjected to MD simulation.

### 3.3 Atomic-level characterization and binding free energy calculations

Characterization of the protein-ligand complex is essential for predicting selective GSK3β inhibitors. The top two FDA-approved drugs (selinexor and ruboxistaurin) were selected for molecular dynamics (MD) simulation based on the highest predicted activity [according to the negative log of the half-maximal inhibitory concentration (pIC_50_) value] to further investigate the structural stability of the protein-ligand complexes. The three-dimensional structure (PDB ID: 5K5N) of GSK3β was retrieved from the Protein DataBank and used to generate multiple docking poses to select the best conformer for the MD simulation.

**Figure 8:**
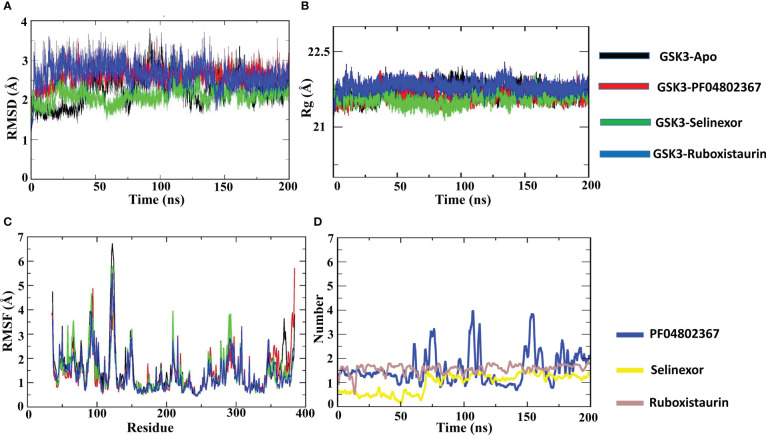
Atomic level characterization. Molecular docking simulation results on apo-GSK3β and complexes of GSK3β with PF04802367, selinexor, and ruboxistaurin. **(A)** Root mean square deviation (RMSD) of the apo-form of GSK3β and the complexes; **(B)** root mean square fluctuations (RMSFs) of the apo-form of GSK3β and the complexes; **(C)** the radius of gyration (Rg) of the apo-form of GSK3β and the complexes; **(D)** Number of hydrogen bonds in the three complexes; PF-04802367, selinexor and ruboxistaurin. (ns:nanoseconds).

All GSK3-apo, PF-04802367 and ligand-bound complexes were docked and subjected to MD simulations in an aqueous environment for 200 nano second (ns) to discern conformational variations and dynamic stability. The dynamic nature of the GSK3β protein and the test drugs (selinexor, ruboxistaurin, and PF-04802367) was explored separately using MD simulation trajectories. To explore the average displacement of the atoms, the root mean squared deviation (RMSD) of the complexes was evaluated and contrasted with the RMSD of the GSK3-apo and PF-04802367 (control) bound structures, as shown in **Figure 8A**. The GSK3β–ruboxistaurin complex showed a stable trajectory with slight variations throughout the simulation, as the RMSD value ranged from 0 to 2.4 Å. However, from 100 ns to 150 ns, the RMSD showed an incremental deviation from 2.5 to 2.7 Å before attaining the final trajectory of 2.4 Å. In the GSK3–selinexor complex, a similar RMSD trajectory profile was observed from 0 to 2.1, and from 50 to 150 ns, with an incremental deviation (2 to 2.4 Å) before reaching 2.1. The RMSD plots for GSK3–PF-04802367 (control drug) showed a steady incremental deviation with the trajectory originating from 0 to 2.8 Å, along with some acceptable variations during different time intervals. The RMSD profile of the apo structure showed a pattern similar to that of GSK3β in complexes with PF-04802367 and ruboxistaurin. However, acceptable deviations were observed in complexes with selinexor compared with those of the apo-form. These findings suggest that after a few conformational changes, the protein-ligand complex achieved a stable conformation during the simulations.

Additionally, to analyze the protein flexibility in the ligand-bound complexes, root mean square fluctuation (RMSF) values were calculated, as shown in **Figure 8B**. The RMSF of the residues measures the residue-level structural fluctuation of a specific segment of the protein that deviates from its mean structure, which often occurs upon ligand binding. The variations observed for each residue represent the degree of flexibility they attained. The ATP-binding site of GSK3β is present at the interface of the N and C termini and consists of Pro136, Leu132, Asp133, Tyr134, and Val135; however, the hydrophobic side chain of Arg141 forms another segment of the pocket (72). In the case of the GSK3β–apo structure, no significant fluctuations occurred in the binding site residues such as Pro136 (0.733 Å), Asp133 (0.633 Å), Tyr134 (0.637 Å), Leu132 (0.806 Å), and Val135 (0.87 Å). In the GSK3–PF04802367 complex, the RMSF values of residues Pro136 (0.614 Å), Leu132 (0.681 Å), Tyr134 (0.584 Å), Val135 (0.547 Å), and Arg141 (0.77 Å) were lower than those found for the apo structure. Similarly, in the GSK3β–selinexor complex, all active-site residues fluctuated less than in the apo structure, particularly residues Pro136 (0.64 Å), Leu132 (0.715 Å), Asp133 (0.593 Å), Tyr134 (0.589 Å), Val135 (0.698 Å), and Arg141 (0.797 Å). The fluctuation patterns of residues Asp133 (0.609 Å) and Tyr134 (0.622 Å) in the GSK3–ruboxistaurin complex were similar to those of the apo structure; however, slightly higher fluctuations were observed for residues Pro136 (0.938 Å) and Arg141 (0.959 Å). The GSK3–ligand interaction reduced the overall fluctuations of the protein; the RMSF value of the selected FDA drugs (selinexor and ruboxistaurin) was similar to that of the control drug PF-04802367.

To further characterize the compactness of the protein in the binding of the ligand at the ATP site of GSK3, the radius of gyration (Rg) was determined, as shown in **Figure 8C**. The impact of ligand binding on the Rg of the GSK3β protein was calculated and compared with that of the apo-GSK3β protein structure. The Rg of the apo and ligand-bound complexes remained between 21.5 and 21.7 Å, which indicated their compactness and sustained stability.

To analyze the formation of hydrogen bonds throughout the MD simulation, the gmx hbond program from the GROMACS package was used. As shown in **Figure 8D**, the average hydrogen bond for the control (PF-04802367) was about 1.5; however, an increase in the number of hydrogen bonds from 1.5 to 3.9 was observed. On the other hand, the average hydrogen bond for selinexor and ruboxistaurin was about 1.3 and 1.6. However, during the early steps (0 to 66 ns) of MD simulation, a low number of hydrogen bonds was observed in selinexor, which was about 0.6. Overall, the hydrogen bond formation remains intact throughout the simulations, indicating that the ligands were present in the binding pocket throughout the process.

**Figure 9:**
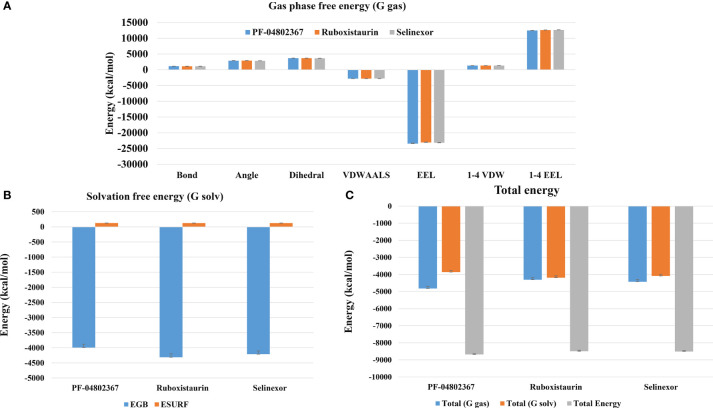
MM-PBSA binding free energy calculation. **(A)** Representative contributions of each Gas phase free energy (G-gas) components for GSK3 with the selected drugs (PF-04802367, ruboxistaurin, and selinexor); **(B)** The solvation free energy for GSK3 in complex with the selected drugs; **(C)** Total of gas phase and solvation free energies along with cumulative total binding free energy for PF-04802367, ruboxistaurin, and selinexor.

The MM-PBSA technique was used to calculate binding free energy to measure the strength of receptor-ligand binding. The change in binding free energy upon binding of ruboxistaurin, selinexor, and PF-04802367 (control) with GSK3 receptors is shown in **Figure 9**. Here, GSK3-drugs (ruboxistaurin, selinexor) displayed acceptable binding free energy, which is comparable with a positive control (PF-04802367). The cumulative binding energy (Gbinding) of selinexor and ruboxistaurin is -8509.006 56.335 kcal/mol and -8491.473 57.039 kcal/mol, whereas PF-04802367 is -8681.320 58.8627 kcal/mol. However, the total binding energy of PF-04802367 is slightly higher but comparable with that of selinexor and ruboxistaurin, which clearly reflects the robustness of our ML model in predicting the drug candidates that could bind tightly to GSK3.

## 4 Conclusion

The COVID-19 pandemic caused by the new coronavirus, SARS-CoV-2, poses a serious threat to the global health system. We employed ML-based predictive modeling to identify FDA-approved and clinical candidate drugs inhibiting GSK3, as this kinase plays a critical role in the phosphorylation of the SARS-CoV-2 N protein that is required for viral replication (16, 20). Furthermore, among the FDA-approved compound libraries, leads with good pIC_50_ values were subjected to MD simulations to investigate protein–drug interactions in a dynamic environment. Recently, one of the identified drugs, selinexor, was also found to be effective against SARS-CoV-2. It is currently in the clinical trial recruitment phase (NCT04534725) for COVID-19 treatment.

We anticipate that the current research, which combines data curation from relevant databases with ML-based predictive algorithms to identify possible therapeutic candidates for COVID-19, could complement ongoing antiviral research efforts. These artificial intelligence–based pipelines may help in the design of preclinical laboratory studies, future clinical trials, and drug discovery. These approaches may also help to improve our understanding of other diseases and related biological phenomena. COVID-19 and potential future outbreaks of coronaviruses may be treatable because of the interference with the conserved dependence of the N protein on GSK3 and its potential role in the regulation of autophagy.

## Data availability statement

The original contributions presented in the study are included in the article/**Supplementary Material**. Further inquiries can be directed to the corresponding author.

## Author contributions

Conceptualization, RP and SC. methodology, RP and BA. Writing—original draft preparation, RP. Writing—review and editing, RP, BA, NQ, and SC. Visualization, BA, and NQ. Supervision, SC. Funding acquisition, SC. All authors contributed to the article and approved the submitted version.

## References

[1] V’kovski P Kratzel A Steiner S Stalder H Thiel V. Coronavirus biology and replication: Implications for SARS-CoV-2. Nat Rev Microbiol (2021) 19:155–70. doi: 10.1038/S41579-020-00468-6 PMC7592455 33116300

[2] Fehr AR Perlman S. Coronaviruses: An overview of their replication and pathogenesis. Methods Mol Biol (2015) 1282:1–23. doi: 10.1007/978-1-4939-2438-7_1 25720466 PMC4369385

[3] Bouhaddou M Memon D Meyer B White KM Rezelj VV Correa Marrero M. The global phosphorylation landscape of SARS-CoV-2 infection. Cell (2020) 182:685–712.e19. doi: 10.1016/J.CELL.2020.06.034 32645325 PMC7321036

[4] Riva L Yuan S Yin X Martin-Sancho L Matsunaga N Pache L. Discovery of SARS-CoV-2 antiviral drugs through large-scale compound repurposing. Nature (2020) 586:113–9. doi: 10.1038/S41586-020-2577-1 PMC7603405 32707573

[5] Garcia G Sharma A Ramaiah A Sen C Purkayastha A Kohn DB. Antiviral drug screen identifies DNA-damage response inhibitor as potent blocker of SARS-CoV-2 replication. Cell Rep (2021) 35. doi: 10.1016/J.CELREP.2021.108940 PMC7969873 33784499

[6] Dittmar M Lee JS Whig K Segrist E Li M Kamalia B. Drug repurposing screens reveal cell-type-specific entry pathways and FDA-approved drugs active against SARS-Cov-2. Cell Rep (2021) 35. doi: 10.1016/J.CELREP.2021.108959 PMC7985926 33811811

[7] Cubuk J Alston JJ Incicco JJ Singh S Stuchell-Brereton MD Ward MD. The SARS-CoV-2 nucleocapsid protein is dynamic, disordered, and phase separates with RNA. Nat Commun (2021) 12. doi: 10.1038/S41467-021-21953-3 PMC8007728 33782395

[8] Chang CK Hou MH Chang CF Hsiao CD Huang TH. The SARS coronavirus nucleocapsid protein–forms and functions. Antiviral Res (2014) 103:39–50. doi: 10.1016/J.ANTIVIRAL.2013.12.009 24418573 PMC7113676

[9] Surjit M Lal SK. The SARS-CoV nucleocapsid protein: A protein with multifarious activities. Infect Genet Evol (2008) 8:397–405. doi: 10.1016/J.MEEGID.2007.07.004 17881296 PMC7106238

[10] Wu CH Chen PJ Yeh SH. Nucleocapsid phosphorylation and RNA helicase DDX1 recruitment enables coronavirus transition from discontinuous to continuous transcription. Cell Host Microbe (2014) 16:462–72. doi: 10.1016/J.CHOM.2014.09.009 PMC7104987 25299332

[11] Wu C Liu Y Yang Y Zhang P Zhong W Wang Y. Analysis of therapeutic targets for SARS-CoV-2 and discovery of potential drugs by computational methods. Acta Pharm Sin B (2020) 10:766–88. doi: 10.1016/J.APSB.2020.02.008 PMC7102550 32292689

[12] Eldar-Finkelman H Eisenstein M. Peptide inhibitors targeting protein kinases. Curr Pharm Des (2009) 15:2463–70. doi: 10.2174/138161209788682253 19601843

[13] Doble BW Woodgett JR. GSK-3: Tricks of the trade for a multi-tasking kinase. J Cell Sci (2003) 116:1175–86. doi: 10.1242/JCS.00384 PMC3006448 12615961

[14] Gould TD Zarate CA Manji HK. Glycogen synthase kinase-3: A target for novel bipolar disorder treatments. J Clin Psychiatry (2004) 65:7849. doi: 10.4088/JCP.v65n0103 14744163

[15] Hur EM Zhou FQ. GSK3 signalling in neural development. Nat Rev Neurosci (2010) 11:539–51. doi: 10.1038/NRN2870 PMC3533361 20648061

[16] Liu X Verma A Garcia G Ramage H Lucas A Myers RL. Targeting the coronavirus nucleocapsid protein through GSK-3 inhibition. Proc Natl Acad Sci U.S.A. (2021) 118. doi: 10.1073/PNAS.2113401118/-/DCSUPPLEMENTAL PMC8594528 34593624

[17] Kaidanovich-Beilin O Woodgett JR. GSK-3: Functional insights from cell biology and animal models. Front Mol Neurosci (2011) 4:40. doi: 10.3389/FNMOL.2011.00040 22110425 PMC3217193

[18] Liang MH Chuang DM. Differential roles of glycogen synthase kinase-3 isoforms in the regulation of transcriptional activation. J Biol Chem (2006) 281:30479–84. doi: 10.1074/JBC.M607468200 16912034

[19] Rao R Patel S Hao C Woodgett J Harris R. GSK3beta mediates renal response to vasopressin by modulating adenylate cyclase activity. J Am Soc Nephrol (2010) 21:428–37. doi: 10.1681/ASN.2009060672 PMC2831860 20056751

[20] Rudd CE. GSK-3 inhibition as a therapeutic approach against SARs CoV2: Dual benefit of inhibiting viral replication while potentiating the immune response. Front Immunol (2020) 11:1638. doi: 10.3389/FIMMU.2020.01638 32695123 PMC7333796

[21] White TC Yi Z Hogue BG. Identification of mouse hepatitis coronavirus A59 nucleocapsid protein phosphorylation sites. Virus Res (2007) 126:139–48. doi: 10.1016/J.VIRUSRES.2007.02.008 PMC2001268 17367888

[22] Calvo E Escors D López JA González JM Álvarez A Arza E. Phosphorylation and subcellular localization of transmissible gastroenteritis virus nucleocapsid protein in infected cells. J Gen Virol (2005) 86:2255–67. doi: 10.1099/VIR.0.80975-0 16033973

[23] Wu CH Yeh SH Tsay YG Shieh YH Kao CL Chen YS. Glycogen synthase kinase-3 regulates the phosphorylation of severe acute respiratory syndrome coronavirus nucleocapsid protein and viral replication. J Biol Chem (2009) 284:5229–39. doi: 10.1074/JBC.M805747200 PMC8011290 19106108

[24] Emmott E Munday D Bickerton E Britton P Rodgers MA Whitehouse A. The cellular interactome of the coronavirus infectious bronchitis virus nucleocapsid protein and functional implications for virus biology. J Virol (2013) 87:9486–500. doi: 10.1128/JVI.00321-13 PMC3754094 23637410

[25] He L Fei DL Nagiec MJ Mutvei AP Lamprakis A Kim BY. Regulation of GSK3 cellular location by FRAT modulates mTORC1-dependent cell growth and sensitivity to rapamycin. Proc Natl Acad Sci U.S.A. (2019) 116:19523–9. doi: 10.1073/PNAS.1902397116 PMC6765302 31492813

[26] Bautista SJ Boras I Vissa A Mecica N Yip CM Kim PK. mTOR complex 1 controls the nuclear localization and function of glycogen synthase kinase 3β. J Biol Chem (2018) 293:14723–39. doi: 10.1074/JBC.RA118.002800 PMC6153275 30061153

[27] Qin X Jiang B Zhang Y. 4E-BP1, a multifactor regulated multifunctional protein. Cell Cycle (2016) 15:781–6. doi: 10.1080/15384101.2016.1151581 PMC4845917 26901143

[28] Sridharan S Basu A. Distinct roles of mTOR targets S6K1 and S6K2 in breast cancer. Int J Mol Sci (2020) 21. doi: 10.3390/IJMS21041199 PMC7072743 32054043

[29] Settembre C Fraldi A Medina DL Ballabio A. Signals from the lysosome: a control centre for cellular clearance and energy metabolism. Nat Rev Mol Cell Biol (2013) 14:283–96. doi: 10.1038/NRM3565 PMC4387238 23609508

[30] Li Y Xu M Ding X Yan C Song Z Chen L. Protein kinase c controls lysosome biogenesis independently of mTORC1. Nat Cell Biol (2016) 18:1065–77. doi: 10.1038/NCB3407 27617930

[31] García-Pérez BE González-Rojas JA Salazar MI Torres-Torres C Castrejón-Jiménez NS. Taming the autophagy as a strategy for treating COVID-19. Cells (2020) 9. doi: 10.3390/CELLS9122679 PMC7764362 33322168

[32] Lakshmana MK. SARS-CoV-2-induced autophagy dysregulation may cause neuronal dysfunction in COVID-19. Neural Regener Res (2022) 17:1255–6. doi: 10.4103/1673-5374.327333 PMC8643044 34782561

[33] Kumar AV Thakurta TG Silvestrini MJ Johnson JR Reenan RA Lapierre LR. Give me a SINE: How selective inhibitors of nuclear export modulate autophagy and aging. Mol Cell Oncol (2018) 5. doi: 10.1080/23723556.2018.1502511 PMC6154834 30263946

[34] Sedykh A Zhu H Tang H Zhang L Richard A Rusyn I. Use of *in vitro* HTS-derived concentration-response data as biological descriptors improves the accuracy of QSAR models of *in vivo* toxicity. Environ Health Perspect (2011) 119:364–70. doi: 10.1289/EHP.1002476 PMC3060000 20980217

[35] Cherkasov A Muratov EN Fourches D Varnek A Baskin II Cronin M. QSAR modeling: Where have you been? where are you going to? J Med Chem (2014) 57:4977–5010. doi: 10.1021/JM4004285 24351051 PMC4074254

[36] Neves BJ Braga RC Melo-Filho CC Moreira-Filho JT Muratov EN Andrade CH. QSAR-based virtual screening: Advances and applications in drug discovery. Front Pharmacol (2018) 9:1275. doi: 10.3389/FPHAR.2018.01275 30524275 PMC6262347

[37] Yan D Lee S Thakkar VD Luo M Moore ML Plemper RK. Cross-resistance mechanism of respiratory syncytial virus against structurally diverse entry inhibitors. Proc Natl Acad Sci U.S.A. (2014) 111. doi: 10.1073/PNAS.1405198111 PMC4143008 25092342

[38] Silva RCMC Ribeiro JS da Silva GPD da Costa LJ Travassos LH. Autophagy modulators in coronavirus diseases: A double strike in viral burden and inflammation. Front Cell Infect Microbiol (2022) 12:845368. doi: 10.3389/FCIMB.2022.845368 35433503 PMC9010404

[39] Jang WD Jeon S Kim S Lee SY. Drugs repurposed for COVID-19 by virtual screening of 6,218 drugs and cell-based assay. Proc Natl Acad Sci U.S.A. (2021) 118. doi: 10.1073/PNAS.2024302118 PMC8325362 34234012

[40] Mendez D Gaulton A Bento AP Chambers J de VM Félix E. ChEMBL: Towards direct deposition of bioassay data. Nucleic Acids Res (2019) 47:D930–40. doi: 10.1093/NAR/GKY1075 PMC6323927 30398643

[41] Fernández-Torras A Comajuncosa-Creus A Duran-Frigola M Aloy P. Connecting chemistry and biology through molecular descriptors. Curr Opin Chem Biol (2022) 66. doi: 10.1016/J.CBPA.2021.09.001 34626922

[42] Shameera Ahamed TK Rajan VK Sabira K Muraleedharan K. QSAR classification-based virtual screening followed by molecular docking studies for identification of potential inhibitors of 5-lipoxygenase. Comput Biol Chem (2018) 77:154–66. doi: 10.1016/J.COMPBIOLCHEM.2018.10.002 30321850

[43] Yap CW. PaDEL-descriptor: an open source software to calculate molecular descriptors and fingerprints. J Comput Chem (2011) 32:1466–74. doi: 10.1002/JCC.21707 21425294

[44] Jović A Brkić K Bogunović N. A review of feature selection methods with applications. In: 2015 38th international convention on information and communication technology, electronics and microelectronics, MIPRO 2015 - proceedings (2015). Opatija, Croatia: IEEE, p. 1200–5. doi: 10.1109/MIPRO.2015.7160458

[45] Ye Q Li M Zhou Y Pang T Xu L Cao J. Synthesis and biological evaluation of 3-benzisoxazolyl-4-indolylmaleimides as potent, selective inhibitors of glycogen synthase kinase-3β. Molecules (2013) 18:5498–516. doi: 10.3390/MOLECULES18055498 PMC6270165 23669633

[46] Heider F Ansideri F Tesch R Pantsar T Haun U Döring E. Pyridinylimidazoles as dual glycogen synthase kinase 3β/p38α mitogen-activated protein kinase inhibitors. Eur J Med Chem (2019) 175:309–29. doi: 10.1016/J.EJMECH.2019.04.035 31096153

[47] Zhang HC White KB Ye H McComsey DF Derian CK Addo MF. Macrocyclic bisindolylmaleimides as inhibitors of protein kinase c and glycogen synthase kinase-3. Bioorg Med Chem Lett (2003) 13:3049–53. doi: 10.1016/S0960-894X(03)00644-9 12941331

[48] Lavecchia A. Machine-learning approaches in drug discovery: methods and applications. Drug Discovery Today (2015) 20:318–31. doi: 10.1016/J.DRUDIS.2014.10.012 25448759

[49] GitHub - shankarpandala/lazypredict at master . Available at: https://github.com/shankarpandala/lazypredict/tree/master (Accessed 9, 2023).

[50] Pirzada RH Haseeb M Batool M Kim M Choi S. Remdesivir and ledipasvir among the FDA-approved antiviral drugs have potential to inhibit SARS-CoV-2 replication. Cells (2021) 10. doi: 10.3390/CELLS10051052 PMC8146643 33946869

[51] Valdés-Tresanco MS Valdés-Tresanco ME Valiente PA Moreno E. gmx_MMPBSA: A new tool to perform end-state free energy calculations with GROMACS. J Chem Theory Comput (2021) 17:6281–91. doi: 10.1021/ACS.JCTC.1C00645 34586825

[52] Ahmad B Choi S. Unraveling the tomaralimab epitope on the toll-like receptor 2 *via* molecular dynamics and deep learning. ACS Omega (2022) 7:28226–37. doi: 10.1021/ACSOMEGA.2C02559 PMC9386714 35990491

[53] Vempati UD Przydzial MJ Chung C Abeyruwan S Mir A Sakurai K. Formalization, annotation and analysis of diverse drug and probe screening assay datasets using the BioAssay ontology (BAO). PloS One (2012) 7. doi: 10.1371/JOURNAL.PONE.0049198 PMC3498356 23155465

[54] Menichetti R Kanekal KH Bereau T. Drug-membrane permeability across chemical space. ACS Cent Sci (2019) 5:290–8. doi: 10.1021/ACSCENTSCI.8B00718/SUPPL_FILE/OC8B00718_SI_003.TXT PMC6396385 30834317

[55] Di L Kerns EH. Lipophilicity. In: Drug-like properties (2016). Boston: Academic Press, p. 39–50. doi: 10.1016/B978-0-12-801076-1.00005-8

[56] Lipinski CA Lombardo F Dominy BW Feeney PJ. Experimental and computational approaches to estimate solubility and permeability in drug discovery and development settings. Adv Drug Delivery Rev (2001) 46:3–26. doi: 10.1016/S0169-409X(00)00129-0 11259830

[57] Gleeson MP. Generation of a set of simple, interpretable ADMET rules of thumb. J Med Chem (2008) 51:817–34. doi: 10.1021/JM701122Q 18232648

[58] Lipinski CA. Lead- and drug-like compounds: The rule-of-five revolution. Drug Discovery Today Technol (2004) 1:337–41. doi: 10.1016/J.DDTEC.2004.11.007 24981612

[59] Piryonesi SM El-Diraby TE. Using machine learning to examine impact of type of performance indicator on flexible pavement deterioration modeling. J Infrastructure Syst (2021) 27:4021005. doi: 10.1061/(ASCE)IS.1943-555X.0000602

[60] Vignaux PA Minerali E Foil DH Puhl AC Ekins S. Machine learning for discovery of GSK3β inhibitors. ACS Omega (2020) 5:26551–61. doi: 10.1021/ACSOMEGA.0C03302/ASSET/IMAGES/LARGE/AO0C03302_0005.JPEG PMC7581251 33110983

[61] Berrouet C Dorilas N Rejniak KA Tuncer N. Comparison of drug inhibitory effects (IC 50) in monolayer and spheroid cultures. Bull Math Biol (2020) 82:1–23. doi: 10.1007/S11538-020-00746-7/TABLES/3 PMC9773863 32495209

[62] Gandhi UH Senapedis W Baloglu E Unger TJ Chari A Vogl D. Clinical implications of targeting XPO1-mediated nuclear export in multiple myeloma. Clin Lymphoma Myeloma Leuk (2018) 18:335–45. doi: 10.1016/J.CLML.2018.03.003 29610030

[63] Mostafa-Hedeab G Al-kuraishy HM Al-Gareeb AI Welson NN Batiha GE-S Conte-Junior CA. Selinexor and COVID-19: The neglected warden. Front Pharmacol (2022) 13:884228. doi: 10.3389/FPHAR.2022.884228 35559257 PMC9086449

[64] Kashyap T Murray J Walker CJ Chang H Tamir S Hou B. Selinexor, a novel selective inhibitor of nuclear export, reduces SARS-CoV-2 infection and protects the respiratory system *in vivo* . Antiviral Res (2021) 192. doi: 10.1016/J.ANTIVIRAL.2021.105115 PMC8213878 34157321

[65] Lear T Larsen M Lin B Cao Q Alfaras I Kennerdell J. Modulation of lysosomal function as a therapeutic approach for coronaviral infections. Res Sq (2021) 36. doi: 10.21203/RS.3.RS-419305/V1

[66] Choi Y Bowman JW Jung JU. Autophagy during viral infection - a double-edged sword. Nat Rev Microbiol (2018) 16:341–54. doi: 10.1038/S41579-018-0003-6 PMC6907743 29556036

[67] Deretic V Saitoh T Akira S. Autophagy in infection, inflammation and immunity. Nat Rev Immunol (2013) 13:722–37. doi: 10.1038/NRI3532 PMC5340150 24064518

[68] Wang C Wang H Zhang D Luo W Liu R Xu D. Phosphorylation of ULK1 affects autophagosome fusion and links chaperone-mediated autophagy to macroautophagy. Nat Commun (2018) 9. doi: 10.1038/S41467-018-05449-1 PMC6113293 30154410

[69] Mao J Lin E He L Yu J Tan P Zhou Y. Autophagy and viral infection. Adv Exp Med Biol (2019) 1209:55–78. doi: 10.1007/978-981-15-0606-2_5 31728865 PMC7122562

[70] Pan HY Valapala M. Regulation of autophagy by the glycogen synthase kinase-3 (GSK-3) signaling pathway. Int J Mol Sci (2022) 23. doi: 10.3390/IJMS23031709 PMC8836041 35163631

[71] Dowey R Cole J Roger Thompson AA Hull RC Huang C Whatmore J. Enhanced neutrophil extracellular trap formation in COVID-19 is inhibited by the protein kinase c inhibitor ruboxistaurin. ERJ Open Res (2022) 8. doi: 10.1183/23120541.00596-2021 PMC8801155 35382002

[72] Liang SH Chen JM Normandin MD Chang JS Chang GC Taylor CK. Discovery of a highly selective glycogen synthase kinase-3 inhibitor (PF-04802367) that modulates tau phosphorylation in the brain: Translation for PET neuroimaging. Angew Chem Int Ed Engl (2016) 55:9601–5. doi: 10.1002/ANIE.201603797 PMC4983481 27355874

