# Supplementary material for: Modeling structure–activity relationships with machine learning to identify GSK3-targeted small molecules as potential COVID-19 therapeutics
Source: Front Endocrinol (Lausanne). 2023 Mar 6;14:1084327. doi: 10.3389/fendo.2023.1084327 (PMC10025526; doi:10.3389/fendo.2023.1084327)
Supplement: Supplementary file 1 [file Table_1.docx]

<Supplementary Material>

Modeling structure–activity relationships with machine learning to identify GSK3-targeted small molecules as potential COVID-19 therapeutics

Rameez Hassan Pirzada^1^, Bilal Ahmad^1^, Naila Qayyum^1^, and Sangdun Choi^1,2*^

^1^Department of Molecular Science and Technology, Ajou University, Suwon 16499, Korea

^2^S&K Therapeutics, Ajou University Campus Plaza 418, 199 Worldcup-ro, Yeongtong-gu, Suwon 16502, Korea

*** Correspondence:**Sangdun Choi
[sangdunchoi@ajou.ac.kr](mailto:sangdunchoi@ajou.ac.kr).

**Supplementary Table S1**. Histogram-based gradient boosting ML (HGBM) model prediction on external dataset were compared with the experimental IC_50_ values.con

| **Pubchem** | **Experimental pIC50** | **HGBM prediction** | **Prediction error** | **Absolute prediction error** | **Squared error** | **Percent error** |
| --- | --- | --- | --- | --- | --- | --- |
| SB 216763 | 8.2 | 7.5 | 0.6 | 0.640 | 0.41 | 7.8% |
| AR-A014418 | 8.7 | 6.5 | 2.2 | 2.218 | 4.92 | 25.5% |
| Tideglusib | 8.3 | 5.3 | 3.0 | 3.032 | 9.19 | 36.5% |
| TWS119 | 7.5 | 8.0 | -0.5 | 0.493 | 0.24 | 6.5% |
| SB415286 | 7.1 | 6.0 | 1.1 | 1.074 | 1.15 | 15.1% |
| AZD2858 | 8.3 | 7.2 | 1.1 | 1.092 | 1.19 | 13.2% |
| PHA-767491 | 6.7 | 4.9 | 1.8 | 1.750 | 3.06 | 26.3% |
| BAY-1143572 | 7.1 | 7.9 | -0.8 | 0.829 | 0.69 | 11.7% |
| CHIR-98014 | 9.2 | 6.2 | 3.0 | 2.986 | 8.92 | 32.4% |
| 1129669-05-1 | 7.3 | 6.4 | 0.9 | 0.912 | 0.83 | 12.5% |
| 667463-85-6 | 8.0 | 5.4 | 2.6 | 2.559 | 6.55 | 32.0% |
| BIO-Acetoxime | 8.0 | 5.4 | 2.6 | 2.559 | 6.55 | 32.0% |
| PF-04 802367 | 9.0 | 6.3 | 2.6 | 2.641 | 6.98 | 29.5% |
| BRD0705 | 7.1 | 6.03 | 1.1 | 1.070 | 1.14 | 15.1% |
| Indirubin | 8.4 | 6.0 | 2.4 | 2.416 | 5.84 | 28.8% |
| LY2090314 | 9.2 | 8.7 | 0.5 | 0.459 | 0.21 | 5.0% |
| AR-A014418 | 8.7 | 6.5 | 2.2 | 2.218 | 4.92 | 25.5% |
| alsterpaullone | 8.4 | 7.8 | 0.6 | 0.616 | 0.38 | 7.3% |
| Kenpaullone | 7.6 | 7.5 | 0.2 | 0.170 | 0.03 | 2.2% |
| SB-415286 | 7.3 | 6.0 | 1.3 | 1.262 | 1.59 | 17.3% |

**Supplementary Table S2**. Light gradient boosting ML (LGBM) model prediction on external dataset were compared with the experimental IC_50_ values.

| **Pubchem** | **Experimental pIC50** | **LGBM prediction** | **Prediction error** | **Absolute prediction error** | **Squared error** | **Percent error** |
| --- | --- | --- | --- | --- | --- | --- |
| SB 216763 | 8.2 | 6.8 | 1.4 | 1.38 | 1.9 | 17.0% |
| AR-A014418 | 8.7 | 5.4 | 3.2 | 3.24 | 10.5 | 37.3% |
| Tideglusib | 8.3 | 5.7 | 2.6 | 2.62 | 6.9 | 31.6% |
| TWS119 | 7.5 | 6.6 | 0.9 | 0.94 | 0.9 | 12.5% |
| SB415286 | 7.1 | 5.8 | 1.3 | 1.31 | 1.7 | 18.5% |
| AZD2858 | 8.3 | 6.6 | 1.7 | 1.71 | 2.9 | 20.6% |
| PHA-767491 | 6.7 | 5.2 | 1.4 | 1.42 | 2.0 | 21.4% |
| BAY-1143572 | 7.1 | 6.6 | 0.5 | 0.49 | 0.2 | 6.9% |
| CHIR-98014 | 9.2 | 6.0 | 3.2 | 3.17 | 10.1 | 34.4% |
| 1129669-05-1 | 7.3 | 6.0 | 1.3 | 1.28 | 1.6 | 17.6% |
| 667463-85-6 | 8.0 | 5.7 | 2.3 | 2.29 | 5.3 | 28.7% |
| BIO-Acetoxime | 8.0 | 5.7 | 2.3 | 2.29 | 5.3 | 28.7% |
| PF-04802367 | 9.0 | 6.1 | 2.8 | 2.81 | 7.9 | 31.4% |
| BRD0705 | 7.1 | 6.5 | 0.6 | 0.60 | 0.4 | 8.5% |
| Indirubin | 8.4 | 6.0 | 2.4 | 2.44 | 5.9 | 29.0% |
| LY2090314 | 9.2 | 7.5 | 1.6 | 1.61 | 2.6 | 17.5% |
| AR-A014418 | 8.7 | 5.4 | 3.2 | 3.24 | 10.5 | 37.3% |
| alsterpaullone | 8.4 | 6.9 | 1.5 | 1.53 | 2.3 | 18.2% |
| Kenpaullone | 7.6 | 6.6 | 1.1 | 1.05 | 1.1 | 13.8% |
| SB-415286 | 8.2 | 5.8 | 1.5 | 1.50 | 2.3 | 20.6% |

**Supplementary Table S3.** Lazy Predict to evaluate and fit all machine learning model.

| Model | Adjusted R-Squared | R-Squared | RMSE | Time Taken |
| --- | --- | --- | --- | --- |
| HistGradientBoostingRegressor | 0.376136524 | 0.514578138 | 0.968098069 | 3.424556732 |
| LGBMRegressor | 0.376136523 | 0.514578138 | 0.96809807 | 0.416507244 |
| NuSVR | 0.335751737 | 0.483155144 | 0.998940882 | 2.465463877 |
| RandomForestRegressor | 0.333605913 | 0.4814855 | 1.000553098 | 5.622033358 |
| SVR | 0.325463513 | 0.475149981 | 1.006647216 | 2.871527433 |
| XGBRegressor | 0.315710582 | 0.467561324 | 1.013898512 | 1.940574169 |
| KNeighborsRegressor | 0.296125256 | 0.45232218 | 1.028305751 | 0.853377819 |
| BaggingRegressor | 0.287547222 | 0.445647698 | 1.034552693 | 0.508792639 |
| GradientBoostingRegressor | 0.253420595 | 0.419094114 | 1.059040519 | 1.653486967 |
| MLPRegressor | 0.233052518 | 0.40324592 | 1.073389608 | 6.038051844 |
| ElasticNetCV | 0.126456354 | 0.320304523 | 1.145557461 | 5.065680981 |
| LassoCV | 0.12632323 | 0.32020094 | 1.145644746 | 4.422708511 |
| RidgeCV | 0.121959795 | 0.316805796 | 1.14850205 | 0.172245026 |
| SGDRegressor | 0.121883663 | 0.316746558 | 1.148551841 | 0.180692673 |
| BayesianRidge | 0.121397916 | 0.316368603 | 1.148869469 | 0.160074949 |
| Ridge | 0.111081707 | 0.308341665 | 1.155594578 | 0.056850433 |
| PoissonRegressor | 0.103009797 | 0.302060994 | 1.160829466 | 0.112086296 |
| LassoLarsIC | 0.100823952 | 0.30036021 | 1.162242998 | 0.172918797 |
| HuberRegressor | 0.098644647 | 0.298664515 | 1.163650592 | 0.954362392 |
| LassoLarsCV | 0.084377683 | 0.287563534 | 1.172823767 | 0.447772264 |
| LinearRegression | 0.065956596 | 0.273230273 | 1.184562834 | 0.101348162 |
| TransformedTargetRegressor | 0.065956596 | 0.273230273 | 1.184562834 | 0.106431723 |
| LinearSVR | 0.036470434 | 0.250287388 | 1.203114875 | 0.897155762 |
| GeneralizedLinearRegressor | 0.022463643 | 0.239388846 | 1.21182814 | 0.076607466 |
| TweedieRegressor | 0.022463643 | 0.239388846 | 1.21182814 | 0.137164593 |
| GammaRegressor | 0.017507056 | 0.235532176 | 1.214896536 | 0.070983887 |
| OrthogonalMatchingPursuitCV | 0.010581741 | 0.230143658 | 1.219170748 | 0.14483428 |
| OrthogonalMatchingPursuit | 0.010581741 | 0.230143658 | 1.219170748 | 0.056981325 |
| ExtraTreesRegressor | -0.005920957 | 0.217303076 | 1.229296094 | 5.544952154 |
| AdaBoostRegressor | -0.006890318 | 0.216548825 | 1.229888261 | 1.061925173 |
| LarsCV | -0.037378763 | 0.192826075 | 1.248369789 | 0.614511013 |
| DecisionTreeRegressor | -0.066082433 | 0.170492039 | 1.26552278 | 0.168985844 |
| ExtraTreeRegressor | -0.077353642 | 0.161722026 | 1.272195092 | 0.127177954 |
| Lasso | -0.287996165 | -0.002176791 | 1.391015145 | 0.063404322 |
| ElasticNet | -0.287996165 | -0.002176791 | 1.391015145 | 0.061010838 |
| DummyRegressor | -0.287996165 | -0.002176791 | 1.391015145 | 0.037137032 |
| LassoLars | -0.287996165 | -0.002176791 | 1.391015145 | 0.07375288 |
| PassiveAggressiveRegressor | -0.635126546 | -0.272275431 | 1.567293232 | 0.108082771 |
| GaussianProcessRegressor | -12.63012631 | -9.60546345 | 4.52506179 | 2.588134289 |
| KernelRidge | -25.54271797 | -19.65262044 | 6.314620713 | 0.84377718 |
| Lars | -770.648638 | -599.412002 | 34.04743274 | 0.149011135 |
| RANSACRegressor | -7.18E+23 | -5.59E+23 | 1.04E+12 | 1.561801672 |

**Supplementary Table S4.** Light gradient boosting ML (LGBM) and Histogram-based gradient boosting ML (HGBM) model prediction for the external dataset.

|  | Name | Our Model | |
| --- | --- | --- | --- |
|  |  | HGBM prediction | LGBM Prediction |
| 1 | Dantrolene | 5.3601398 | 6.216508127 |
| 2 | Darifenacin | 5.700038 | 5.977120852 |
| 3 | Nefopam | 4.0606809 | 4.927278097 |
| 4 | Nifedipine | 5.5558209 | 5.422156862 |
| 5 | Nithiamide | 6.9182758 | 5.715783347 |
| 6 | Ruboxistaurin | 7.4198136 | 7.448867965 |
| 7 | Thiabendazole | 5.774662 | 5.483706978 |
